# Phase I study of epirubicin, cisplatin and capecitabine plus matuzumab in previously untreated patients with advanced oesophagogastric cancer

**DOI:** 10.1038/sj.bjc.6604622

**Published:** 2008-09-02

**Authors:** S Rao, N Starling, D Cunningham, M Benson, A Wotherspoon, C Lüpfert, R Kurek, J Oates, J Baselga, A Hill

**Affiliations:** 1 Department of Medicine, Royal Marsden Hospital, Surrey, UK; 2 Merck KGaA, Darmstadt, Germany; 3 University of Hebron, Barcelona, Spain

**Keywords:** oesophagogastric cancer, advanced, chemotherapy, anti-EGFR therapy, ECX

## Abstract

To evaluate the safety, tolerability, efficacy, pharmacokinetics and pharmacodynamics of the humanised antiepidermal growth factor receptor monoclonal antibody matuzumab combined with epirubicin, cisplatin and capecitabine (ECX) in patients as first-line treatment for advanced oesophagogastric cancer that express epidermal growth factor receptor (EGFR). This was a phase I dose escalation study of matuzumab at 400 and 800 mg weekly and 1200 mg every 3 weeks combined with ECX (epirubicin 50 mg m^−2^, cisplatin 60 mg m^−2^ on day 1 and capecitabine 1000 mg m^−2^ daily). Patients were treated until disease progression, unacceptable toxicity or for a maximum of eight cycles. Twenty-one patients were treated with matuzumab at three different dose levels (DLs) combined with ECX. The main dose-limiting toxicity (DLT) was grade 3 lethargy at 1200 mg matuzumab every 3 weeks and thus 800 mg matuzumab weekly was the maximum-tolerated dose (MTD). Other common toxicities included rash, nausea, stomatitis and diarrhoea. Pharmacokinetic evaluation demonstrated that the coadministration of ECX did not alter the exposure of matuzumab. Pharmacodynamic studies on skin biopsies demonstrated inhibition of the EGFR pathway. Objective response rates of 65% (95% confidence interval (CI): 43–82), disease stabilisation of 25% (95% CI: 11–47) and a disease control rate (CR+PR+SD) of 90% were achieved overall. The MTD of matuzumab in combination with ECX was 800 mg weekly, and at this DL it was well-tolerated and showed encouraging antitumour activity. At the doses evaluated in serial skin biopsies, matuzumab decreased phosphorylation of EGFR and MAPK, and increased phosphorylation of STAT-3.

Oesophagogastric (OG) cancer represents a major health burden worldwide (Parkin, 2001). For patients with advanced disease combination, chemotherapy has shown a survival benefit compared to best supportive care (Murad *et al*, 1993; Pyrhonen *et al*, 1995).

ECF (epirubicin, cisplatin, infused 5FU) is the reference regimen in the United Kingdom and other parts of Europe for advanced OG cancer based on superior response rates, survival and global QOL in several phase III studies (Webb *et al*, 1997; Waters *et al*, 1999; Ross *et al*, 2002). Furthermore, a recent meta-analysis concluded that the best survival results are achieved with regimens containing anthracyclines, cisplatin and 5FU and among these ECF was the most well-tolerated (Wagner *et al*, 2006).

Recently, the V325 study demonstrated a survival benefit for TCF (docetaxel, cisplatin and 5FU) *vs* CF (cisplatin and 5FU) although TCF was associated with >80% grade 3 and 4 neutropaenia (Van Cutsem *et al*, 2006). The randomised phase III trial REAL-2 evaluated four treatment arms ECF, EOF, ECX and EOX (E, epirubicin; X, capecitabine; C, cisplatin; O, oxaliplatin; F, 5FU). Non-inferiority was demonstrated for capecitabine *vs* 5FU and oxaliplatin *vs* cisplatin with acceptable toxicity for all treatment arms (Cunningham *et al*, 2008).

Despite recent advances, the median overall survival with combination chemotherapy is approximately 10–11 months, thus newer treatment strategies are required. The epidermal growth factor receptor (EGFR) has previously been identified as a novel target for anticancer treatment. Epidermal growth factor receptor activation leads to a cascade of signal transduction pathways involved in cell proliferation, angiogenesis, metastasis and invasion (Kim and Muller, 1999; Olayioye *et al*, 1999; Sako *et al*, 2000; Schlessinger, 2000). In oesophageal cancer, expression of EGFR has been reported to be 80–90% (Itakura *et al*, 1994) and is associated with poorer survival.

Matuzumab is a humanised antibody that competitively inhibits natural ligand binding to the EGF receptor with abrogation of EGFR downstream signalling. Matuzumab has also shown antibody-dependent cellular cytotoxicity in these models (Bier *et al*, 1998). Antitumour activity of matuzumab has been observed in non-clinical xenograft models (Amendt *et al*, 2003; Burger *et al*, 2003).

In a phase I study of matuzumab monotherapy in solid tumours, grade 3 headache was identified as the main dose-limiting toxicity (DLT) at 2000 mg weekly, the maximum-tolerated dose (MTD) was 1600 mg weekly and antitumour activity was seen in one heavily pre-treated oesophageal cancer patient (Vanhoefer *et al*, 2004). In recent phase I studies of chemotherapy plus matuzumab in lung and pancreatic cancer (at doses ranging from 100 to 800 mg weekly), the MTD was not reached although one DLT of grade 4 neutropaenia was observed at matuzumab 800 mg combined with paclitaxel. Antitumour activity was reported and pharmacodynamic data revealed blockade of the EGFR pathway (Graeven *et al*, 2006; Kollmannsberger *et al*, 2006). Preliminary data of the phase I study of PFL (cisplatin, leucovorin and 5FU) and matuzumab (at doses of 400 or 800 mg weekly) in advanced OG cancer indicate good tolerability at the 400 mg dose level (DL) (Trarbach *et al*, 2005).

The primary objective of this phase I study was to determine the MTD, recommended dose (RD), safety, tolerability, pharmacokinetic and pharmacodynamic profile of matuzumab combined with ECX in advanced OG tumours expressing EGFR.

## Patients and methods

### Study design

This phase I open label study was divided into two parts. The first two cycles were designated as phase A, to determine the MTD, pharmacokinetic and pharmacodynamic parameters. In phase B, matuzumab plus ECX was continued on the DL selected in phase A until disease progression, unacceptable toxicity or for a further six cycles. Patients who did not complete phase A for any reason, except unacceptable toxicities or progressive disease, were replaced.

### Patients

Eligibility requirements included histologically confirmed adenocarcinoma of the stomach or lower third of the oesophagus, locally advanced, metastatic or recurrent disease, measurable disease by computed tomography (CT), EGFR expression in tumour tissue, normal cardiac function defined by left ventricular ejection fraction, Karnofsky performance status (KPS) ⩾60%, life expectancy >12 weeks, no prior chemotherapy at all, no radiotherapy or major surgery within 4 weeks before the first study treatment, adequate baseline bone marrow and liver function, a glomerular filtration rate >60 ml min^−1^, no severe uncontrolled comorbidities and signed informed consent.

The study protocol was approved by the local ethics committee and was carried out according to the Declaration of Helsinki and good clinical practice guidelines. The subjects' informed consent was obtained before any study-related activities.

### EGFR expression

Tumour material was obtained from the initial tumour resection or diagnostic biopsy. Epidermal growth factor receptor expression was determined by a central pathologist in representative paraffin-embedded tumour blocks using EGFR pharmDx test kit (from DakoCytomation KGaA, Darmstadt, Germany) as previously reported. Tumours were considered positive if any membrane staining was observed. Only patients with EGFR-positive tumours were enrolled. All assessments were performed and reviewed centrally.

### Pre-treatment and evaluation

Pre-treatment evaluation consisted of medical history, physical examination, full blood count (FBC), serum biochemistry, serum tumour marker, urine analysis, CT scans of the chest abdomen pelvis, multiple gated acquisition scan and chest X-ray. During treatment, monitoring included clinical toxicities assessment, FBC, serum biochemistry and physical examination weekly. Computed tomography scans were performed at weeks 6 and 12 and at the end of the treatment. Flectrocardiogram (ECG), KPS assessment and FBC and biochemistry were repeated at the end of the treatment.

### Administration and dose escalation

Matuzumab was supplied by Merck (Germany) as a lyophilisate of 200 mg per vial. Matuzumab was administered as a 1-h intravenous infusion without premedication in 250 ml of 0.9% normal saline solution. ECX comprised of epirubicin 50 mg m^−2^ given as a 15-min infusion, cisplatin 60 mg m^−2^ given as a 4-h infusion on day 1 and capecitabine 500 mg m^−2^ twice daily given continuously, each cycle duration being 3 weeks. Pre-medication and hydration were administered as described previously (Sumpter *et al*, 2005).

Initially, the study was planned with two DLs of matuzumab 400 and 800 mg weekly combined with ECX. However, an amendment was made to the protocol after pharmacodynamic data from a phase I matuzumab monotherapy study revealed that 1200 mg three weekly was the target effective dose. This provided a strong rationale for extending the dose regimen from weekly to a three-weekly schedule (Tabernero *et al*, 2003). Thus two additional DLs of matuzumab 1200 and 1600 mg, administered every 3 weeks with ECX, were included (Table 1).

No intrasubject dose escalation was performed. At each DL, six patients were initially enrolled. If ⩽1 of the patients experienced a DLT during the first two cycles, the next cohort of patients was treated at the subsequent DL. If ⩾2 of 6 patients at one DL experienced any DLT, additional patients were enrolled at the same DL. The MTD and RD were defined as the DL at which not more than one of six patients experienced a DLT.

### Evaluation of toxicities and response

Toxicities were evaluated weekly and graded according to the National Cancer Institute Common Toxicity Criteria (NCI-CTC; version 2.0). The MTD was based on DLTs observed during the first two cycles. Dose-limiting toxicity was defined as follows: an adverse event related to matuzumab including any grade 3/4 non-haematological toxicities (excluding alopaecia, nausea, vomiting and skin reactions), grade 4 nausea, vomiting and skin reactions and toxicity-related discontinuation of treatment for more than 1 week.

Tumour response was measured by CT scans according to RECIST criteria using unconfirmed responses (Therasse *et al*, 2000). Progression-free survival (PFS) was defined as the interval between the date of administration of the first infusion and the confirmation of progressive disease or death, depending on which occurred first.

### Pharmacokinetics

For pharmacokinetic analysis, blood samples were taken before and 1, 2, 5, 48, 96, 168 and 336 h after the start of the matuzumab infusion in weeks 1 and 4. Serum samples were obtained and handled as previously described (Vanhoefer *et al*, 2004). Concentrations of matuzumab were measured in serum using a validated ELISA. On the basis of the resulting concentrations, PK parameters were calculated by compartmental and non-compartmental standard methods using the software package KINETICA™, version 4.1.1.

### Pharmacodynamics

Normal skin tissue biopsies from the upper arm at the posterior surface were taken before the first cycle, after the second cycle and fourth cycle. Tumour biopsies were taken by endoscopy as part of the routine staging before the first cycle, at the end of the second and fourth cycles. The percentage of cells staining positive for proteins on skin and tumour biopsies were determined as biological markers of the treatment. These pharmacodynamic markers comprised phosphorylated EGFR (p-EGFR), phosphorylated p42/44 MAP kinase (p-MAPK), EGFR, Ki67, p27, phosphorylated STAT3 (p-STAT3) and cytokeratin 1. In addition, phosphorylated protein kinase B was measured in tumour biopsies only. The samples were prepared and investigated as previously described (Albanell *et al*, 2002).

## Results

Between 2002 and 2005, 45 patients underwent EGFR testing for the study at the Royal Marsden Hospital, UK and 60% exhibited positive EGFR expression by immunohistochemical analysis. In total, 21 patients with EGFR-positive tumours received study treatment (Table 1). Baseline patient characteristics are shown in Table 2.

No DLT was observed in the initial cohort. At the 800 mg matuzumab DL, one patient experienced a DLT of grade 3 hypotension. One patient was replaced at each DL as per protocol. At the next DL of matuzumab 1200 mg every 3 weeks, 1 DLT of grade 3 pancreatitis and 1 DLT of grade 3 abdominal pain were reported; however, the DLT of grade 3 lethargy occurred in three of six patients, indicating that the MTD was exceeded. Thus the main DLT was grade 3 lethargy, and inconsistent with our previous experience with ECX chemotherapy, therefore, one further patient was entered at this DL. However, this patient experienced the same DLT, thus four of seven patients experienced the main DLT of grade 3 lethargy. Hence, the DL of 800 mg matuzumab weekly combined with ECX was defined as the MTD and the RD.

During phase B, the median number of cycles of treatment was five for the first two cohorts and three for the 1200 mg matuzumab DL. Among the most frequent toxicities observed were diarrhoea, nausea, vomiting and stomatitis, which can be associated with ECX chemotherapy (Table 3). The most significant matuzumab-related side effects (grades 1–4) across all DLs were lethargy and rash affecting 11 and 13 patients, respectively.

One patient with a recurrent anastomotic OGJ tumour treated at the first DL developed an oesophagobronchial fistula and died subsequent to aspiration pneumonia. The patient had received one dose of matuzumab (400 mg per week) and ECX; this event was deemed unrelated to study treatment.

### Efficacy

Although efficacy was not a primary objective, 20 patients were assessable for tumour response. Thirteen of 20 patients achieved a partial response resulting in an overall objective response rate (ORR) of 65% (95% confidence interval (CI): 43–82). Five patients (25%) (95% CI: 11–47) demonstrated disease stabilisation and two (10%) developed progressive disease. The ORR according to DL is shown in Table 4.

One patient with a T3N1 gastric tumour was downstaged to T2N0 on endoscopic ultrasound after five cycles of ECX plus matuzumab (400 mg per week) and underwent surgical resection followed by post-operative ECX plus matuzumab.

The overall median time to disease progression was 5.2 months (95% CI: 3.0–16.0).

Pharmacokinetic analysis parameters are shown in Table 5. Maximum serum concentrations *C*_max_ were achieved on average 1–2 h after the end of the infusion. The mean values for *C*_max_ for all three DLs ranged between 154 and 442 *μ*g ml^−1^ in week 1 and were dose proportional. The AUC results in the first week AUC (0_-168_) were also approximately dose proportional.

**Figure 1:**
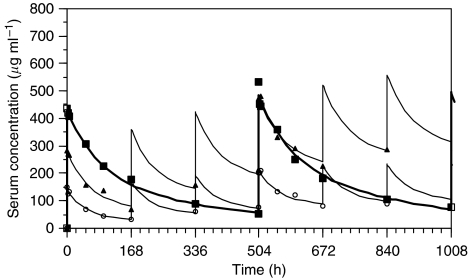
Concentration–time courses of matuzumab in the dose groups 400 mg weekly (dashed line and circles), 800 mg weekly (continuous line and triangles) and 1200 mg every 3 weeks (bold line and squares). The mean concentrations (symbols) are fitted with a two-compartment model per dose group.

The terminal elimination phase was best characterised in the 1200 mg dose group where the concentration–time profile could be assessed over a 3-week period after each matuzumab infusion. Mean terminal elimination half-lives determined after weeks 1 and 4 were in the range of 8–9 days. At lower weekly dosing, only apparent *t*_1/2_ were determined with values of 5–7 days. The mean values for the volume of distribution were consistently small (∼4 l) and dose independent. The mean trough values increased over time towards the steady state (Figure 1). There was evidence of accumulation at the 800 mg weekly DL; there was no correlation between the incidence of DLTs and *C*_max_ and AUC.

### Pharmacodynamics

**Figure 2:**
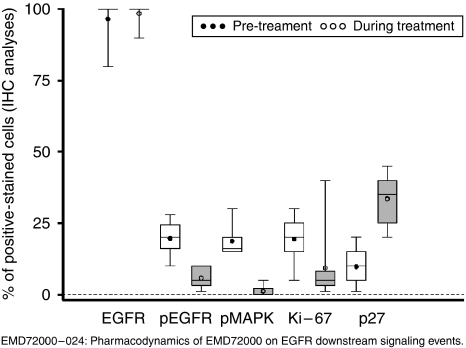
Pharmacodynamics of matuzumab on EGFR downstream signalling events.

Pharmacodynamic results were available from 18 subjects in total. Skin biopsy data were consistent between patients at each DL (Figure 2). Following the administration of matuzumab, the total EGFR expression remained in the range of 80–100%. In contrast, EGFR phosphorylation was inhibited and there was a similar reduction in pMAPK for all investigated DLs. Increased levels of p27 and p-STAT3 were detected. Baseline Ki67 decreased following matuzumab treatment in all but one patient. Cytokeratin 1 levels in skin biopsies generally increased during treatment although there were decreased levels in two patients. The changes in these marker proteins described were not dose dependent.

Tumour biopsy data were available for 15 pre-treatment biopsies but limited for the treatment samples due to poor fixation of tumour tissue. This reflects the difficulties in sampling tumour tissue after the administration of chemotherapy. Thus it was not possible to evaluate the changes observed during treatment in tumour biopsies.

Further investigations were performed to evaluate any correlation between the development of rash and PD changes particularly in the skin biopsies. It was not possible to identify a clear correlation between the change of any PD marker protein in skin or tumour biopsies during matuzumab therapy and the presence of skin rash or response outcome.

## Discussion

This study has demonstrated that ECX combined with matuzumab weekly at doses up to 800 mg per week were generally well-tolerated. At 1200 mg of matuzumab three weekly, the MTD was exceeded and the main DLT experienced by four of seven patients was grade 3 lethargy. This occurred after the first infusion and generally took several weeks to resolve completely. All four patients were of KPS 90 before commencing treatment.

The interim analysis of the REAL-2 study reported 9% grade 3/4 lethargy for patients treated with ECX (Sumpter *et al*, 2005). Asthenia has previously been reported with cetuximab (a chimeric anti-EGFR antibody) in colorectal cancer (CRC). Abubakr *et al* (2006) observed 25% asthenia (6% grade 3/4) in the phase III EPIC study of irinotecan plus or minus cetuximab in 783 patients with refractory CRC. Schrag *et al* (2005) described a magnesium-wasting syndrome associated with severe fatigue-affecting patients with CRC treated with cetuximab and irinotecan. They recommended that serum magnesium levels be monitored for any patient with severe asthenia following administration of cetuximab. In this study (initiated before this publication), the lethargy observed did not appear to be associated with serum hypomagnesaemia, although magnesium was not routinely measured. Thus the mechanism of the fatigue remains unknown but may be due to an interaction between matuzumab at 1200 mg three weekly and ECX chemotherapy.

No allergic reactions, other severe or unexpected adverse events were observed. NCI-CTC grade 1/2 rash was observed in 61.9% of patients in total, which is similar to that previously observed in a phase I study of matuzumab monotherapy (Vanhoefer *et al*, 2004). However, the incidence of rash appears lower than reported with other anti-EGFR monoclonal antibodies including cetuximab or panitumumab, which range between 80 and 90% for all grades (Cunningham *et al*, 2004; Malik *et al*, 2005). The skin toxicity associated with anti-EGFR antibodies is commonly described as an acneiform rash but pathologically resembles an infectious folliculitis (Lenz, 2006) and its pathophysiological mechanism remains unclear.

The pharmacokinetic analyses demonstrated a dose-proportional increase of AUC and *C*_max_ for matuzumab with accumulation suggesting linear pharmacokinetics within the dose range tested. There was no correlation between the incidence of the DLT of lethargy and *C*_max_ and AUC in the 1200 mg cohort. In addition, when comparing the *C*_max_ and AUC_0–168_ of matuzumab plus ECX to a previous matuzumab monotherapy study (Vanhoefer *et al*, 2004), it was found that the matuzumab exposure was similar. This suggests that the coadministration of ECX chemotherapy did not influence the pharmacokinetics of matuzumab.

The PD results obtained from the skin samples were as expected and total EGFR expression was not altered; there was a decrease in p-EGFR, p-MAPK and Ki67, whereas p27 and p-STAT3 increased following the administration of matuzumab. Thus, overall, there was abrogation of EGFR downstream signalling and there was no dose–response relationship. Therefore, there is inhibition of the EGFR network at doses of matuzumab below the MTD. Similar findings have been reported utilising varying schedules of matuzumab (Tabernero *et al*, 2003; Vanhoefer *et al*, 2004). It is not possible in this study to comment on the use of skin as a surrogate for tumour, given the limitations of the tumour biopsies.

There has been much controversy surrounding EGFR expression by immunohistochemistry and the use of anti-EGFR therapy. In this study, all patients were EGFR-positive according to immunohistochemistry; however, there was no apparent correlation between the degree of EGFR inhibition and objective response.

Although efficacy was not a primary objective, only patients with measurable disease were included in the study. The unconfirmed ORR with the combination of ECX plus matuzumab was 65 with 25% disease stabilisation, that is a disease control rate (CR, PR+SD) of 90%, and the median PFS was 5.2 months. The unconfirmed ORR for ECX in the REAL-2 study was 46.4% (Cunningham *et al*, 2008); hence there may be a synergistic or additive effect between matuzumab and ECX although no conclusions can be drawn from these data and further investigation is required in a randomised study. The median time to progression in this study was 5.2 months and thus shorter than that reported for all treatment arms in REAL-2 (Cunningham *et al*, 2008). This may partly be accounted for by the 1200 mg matuzumab cohort who received only five median cycles of treatment and the dose delays incurred before restarting chemotherapy by those patients who experienced the main DLT of grade 3 lethargy.

Several phase I and II studies of EGFR tyrosine kinase inhibitors as immunotherapy in previously treated OG cancers have been reported with response rates ranging from 2.85 to 12% (Ferry *et al*, 2004; Van Groeningen and Giaccone, 2004; Janmaat *et al*, 2006). A phase II study of cetuximab combined with FOLFIRI in untreated gastric and OGJ cancers achieved an ORR of 44.1% (95% CI 27.5–60.9%) (Pinto *et al*, 2007). Janmaat *et al* (2006) identified female gender, squamous histology and high EGFR expression to be associated with improved outcome following the administration of gefitinib in patients with advanced OG cancer. A study of erlotinib demonstrated activity in OGJ tumours but no objective responses in gastric cancers (Dragovich *et al*, 2006). In this trial, the inclusion criteria stipulated adenocarcinoma and the responses to ECX and matuzumab were observed in oesophageal, OGJ and gastric tumours.

In conclusion, this trial has demonstrated that the MTD of matuzumab in combination with ECX was 800 mg weekly and at this DL it was generally well tolerated. Grade 3 lethargy was the main DLT at 1200 mg three weekly and the mechanism for this remains unclear. The combination regimen was associated with clinically meaningful tumour response and stabilisation and the PD markers in skin reflected inhibition of the EGFR signalling at all DLs. Thus a randomised national multicentre phase II trial of ECX with or without the addition of matuzumab at 800 mg weekly in advanced untreated OG cancer has been conducted.

## Figures and Tables

**Table 1:** Study design and dose escalation

**Dose level**	**Matuzumab absolute dose^a^**	**Planned no. of patients**
1	400 mg per week	6
2	800 mg per week	6
3	1200 mg per 3 weeks	6
4	1600 mg per 3 weeks	6

ECX=epirubicin, cisplatin and capecitabine.

a Administered in combination with fixed-dose ECX.

**Table 2:** Patient demographics

**Characteristic**	**Total (*N*=21)**	**%**
*Gender*		
Male	16	76.2
Female	5	23.8
		
*Age (years)*		
Median	59	
		
*Karnofsky performance status*		
60	1	4.8
70	2	9.5
80	5	23.8
90	13	61.9
		
*Primary tumour location*		
Lower one-third of oesophagus	5	23.8
OGJ	7	33.3
Gastric	9	42.9
Adenocarcinoma	21	100
		
*Stage (AJCC)*		
IIIb	1	5
IV	20	95

ECX=epirubicin, cisplatin and capecitabine; OGJ=oesophagogastric junction.

**Table 3:** NCI-CTC all grade toxicities

	**ECX+matuzumab 400 mg weekly *N*=7**				**ECX+matuzumab 800 mg weekly *N*=7**				**ECX+matuzumab 1200 mg every 3 weeks *N*=7**				**Adverse events**	
	**NCI-CTC grade**				**NCI-CTC grade**				**NCI-CTC grade**				**Total**	**(%)**
Toxicity	1	2	3	4	1	2	3	4	1	2	3	4		
Rash	1	2	0	0	4	0	1	0	2	3	0	0	13	61.9
Headache	0	0	0	0	0	0	0	0	1	0	0	0	1	4.8
Diarrhoea	5	0	0	0	3	1	0	0	2	1	2	0	14	66.7
Abdo pain	1	1	0	0	1	0	2	0	0	1	2	0	8	38
PPE	0	1	2	0	0	2	1	0	0	0	0	0	6	28.6
Stomatitis	3	2	0	0	2	2	0	0	5	1	0	1	16	76.2
Nausea	3	2	1	0	2	1	0	0	2	2	3	0	16	76
Vomiting	3	2	1	0	0	0	1	0	2	4	1	0	14	66.7
Lethargy	1	1	0	1	2	1	2	1	0	1	6Ψ	0	16	76
Neutropaenia		0	0	1		0	3	1		1	2	4	12	57
Febrile neutropaenia	0	0	0	0	0	0	0	0	0	0	0	1	1	4.8
Thrombocytopaenia	0	0	1	0	0	0	0	0	0	0	0	0	1	4.8

ECX=epirubicin, cisplatin and capecitabine; NCI-CTC=National Cancer Institute Common Toxicity Criteria; Ψ=Main DLT.

**Table 4:** Objective response to ECX plus matuzumab at all dose levels

	**400 mg matuzumab**	**800 mg matuzumab**	**1200 mg matuzumab**	**Total**
**Dose level**	**weekly *N*=7^a^**	**weekly *N*=7**	**every 3 weeks *N*=7**	***N*=21^a^**
**Response**	***n* (%)**	***n* (%)**	***n* (%)**	***n* (%)**
Partial response (PR)	4 (66.7 [30–90])	3 (42.8 [16–75])	6 (85.7 [49–97])	13 (65 [43–82])
Stable disease (SD)	2 (33.3 [10–70])	2 (28.6 [8–64])	1 (14.3 [3–51])	5 (25 [11–47])
Tumour growth control (PR+SD)	6 (100 [61–100])	5 (71.4 [36–92])	7 (100 [65–100])	18 (90 [70–97])
Progressive disease (PD)	0 (0 [0–39])	2 (28.6 [8–64])	0 (0 [0–35])	2 (10 [3–30])

ECX=epirubicin, cisplatin and capecitabine.

a Response data missing from one patient.

The values inside square brackets denote 95% confidence interval.

**Table 5:** Pharmacokinetic parameters of matuzumab derived by non-compartmental analysis

	**Week 1**						**Week 4**					
	**400 mg per week**		**800 mg per week**		**1200 mg per 3 weeks**		**400 mg per week**		**800 mg per week**		**1200 mg per 3 wk**	
*C*_max_, *μ*g ml^−1^	154 (44)	7	294 (89)	7	442 (108)	7	224 (54)	6	495 (166)	6	534 (125)	6
*t*_max_, h	2.04 (1.05)	7	1.88 (1.45)	7	1.61 (0.55)	7	3.91 (1.81)	6	3.88 (1.88)	6	1.68 (1.63)	6
AUC_*τ*_, *μ*g ml^−1^ h^−1^	10717 (1553)	6	23347 (8748)	6	79189 (19217)	5	19674 (4720)	5	52797 (20448)	5	94868 (22029)	5
AUC_0–∞_ a, *μ*g ml^−1^ h^−1^	12721 (3519)	6	35406 (10593)	7	88066 (24277)	7	NA		NA		NA	
*t*_1/2_, h	80.5 (15)	6	110.8 (36.2)	7	189.5 (23.2)	7	131.4 (31.1)	6	165 (35)	6	221.3 (70.8)	6
CL, h^−1^	0.034 (0.0115)	6	0.0243 (0.0069)	7	0.0145 (0.0038)	7	0.0214 (0.0054)	5	0.0170 (0.0061)	5	0.0131 (0.0025)	5
*V*^b^, l	3.64 (0.53)	6	3.76 (1.51)	7	3.83 (1.14)	7	3.92 (0.44)	5	3.43 (0.96)	5	4.64 (1.22)	5

a AUC_0–*∞*_ is not applicable (NA) for week 4.

b The values reported are *V*_ss_ for week 1 and *V*_Z_ for week 4.

The symbols not explained in the text are *t*_max_, time of *C*_max;_ AUC_*τ*_/AUC_0−*∞*_, AUC within one dosing interval/from time 0 to infinity after single administration; CL, clearance; *V* (*V*_ss_/*V*_Z_), volume of distribution (at steady state/in the terminal phase).

Mean (±s.d.) is given, together with the number of underlying values in the adjacent column.
